# An expedient and new synthesis of pyrrolo[1,2-*b*]pyridazine derivatives

**DOI:** 10.3762/bjoc.5.66

**Published:** 2009-11-17

**Authors:** Rajeshwar Reddy Sagyam, Ravinder Buchikonda, Jaya Prakash Pitta, Himabindu Vurimidi, Pratap Reddy Padi, Mahesh Reddy Ghanta

**Affiliations:** 1 Integrated Product Development, Dr. Reddy’s Laboratories Limited, Bachupally, Qutubullapur, Ranga Reddy District-500072, Andhra Pradesh, India; 2 Institute of Science and Technology, Center for Environmental Science, J. N. T. University, Kukatpally, Hyderabad-500 072, Andhra Pradesh, India; 3 Asha Laboratories, Plot # 175/1, Prashanthi Nagar, Kukatpally, Hyderabad-500072, Andhra Pradesh, India

**Keywords:** 1,4-diketone, migration and cyclization, pyrrolo[1,2-*b*]pyridazine, tertiary butyl carbamate, tertiary butyl carbazate, α,β-unsaturated ketone

## Abstract

The reaction of 2-[2-(4-fluorophenyl)-2-oxo-1-phenylethyl]-4-methyl-3-oxo-pentanoic acid phenylamide with tertiary butyl carbazate and subsequent condensation of the resulting carbamate derivative with a chalcone provided a facile new approach to pyrrolo[1,2-*b*]pyridazine derivatives.

## Introduction

Pyrrolopyridazine derivatives have various biological applications [1–8], and their fluorescent properties have been investigated for potential use in sensors, lasers, and semiconductor devices [9–13].

The synthesis and properties of pyrrolo[1,2-*b*]pyridazine derivatives were reviewed in 1977 by Kuhla and Lombardino [14]. Subsequently, new methods for the synthesis of these compounds have been described, which can be classified into two main approaches. The first involves condensation reactions, such as the condensation of oxazolo[3,2-*b*]pyridazinium perchlorates with malononitrile, ethyl cyanoacetate and ethyl malonate in the presence of sodium ethoxide [15]; the condensation of 1,4,7-triketones with hydrazine followed by dehydrogenation [16]; the condensation of cyanoacetic acid hydrazide with 3-bromo-1,1,3-tricyano-2-phenylpropene [17]; and the reaction between 3-chloropyridazines with propargylic alcohol in the presence of Pd(PPh_3_)_2_Cl_2_–CuI with diethylamine as the reaction medium [18–19]. The second approach is based on cycloaddition reactions, such as the cycloaddition of dimethyl acetylenedicarboxylate to the Reissert compound of pyridazine [20], the 1,3-dipolar cycloaddition of pyridazinium dichloromethylide generated by the carbene method [21], and the cycloaddition of alkylidene cyclopropane derivatives to pyridazine in the presence of Pd(PPh_3_)_4_ [22].

**Figure 1:**
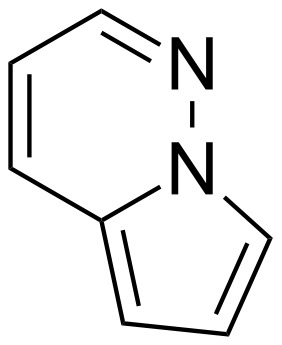
Unsubstituted pyrrolo[1,2-*b*]pyridazine.

Pyrrolo[1,2-*b*]pyridazine derivatives can also be synthesized from 1-aminopyrrole and its derivatives. This original method was reported by Flitsch and Krämer (in 1968–9) [23–24], who obtained a series of unsubstituted pyrrolopyridazines from 1-aminopyrrole and β-dicarbonyl compounds. Benzoylacetone, on condensation with 1-aminopyrrole, forms only one isomer, 2-methyl-4-phenyl-pyrrolopyridazine, whereas benzoylacetaldehyde yields a mixture of 2-phenyl- and 4-phenyl-pyrrolopyridazine. 3-Phenylpyrrolopyridazine is obtained from phenylmalonaldehyde and 1-aminopyrrole [25]. Unsubstituted pyrrolopyridazine (Figure 1) was synthesized in 21% yield from 1-aminopyrrole and 3-ethoxyacrolein diethylacetal [26].

As a part of our continued interest in the development of new synthetic methods for highly substituted pyrrole and indole derivatives [27–28], we have developed a new synthetic route to pyrrolo[1,2-*b*]pyridazines through a hitherto unprecedented approach from a BOC-protected 1-aminopyrrole derivative and α,β-unsaturated ketones.

## Results and Discussion

**Scheme 1:**
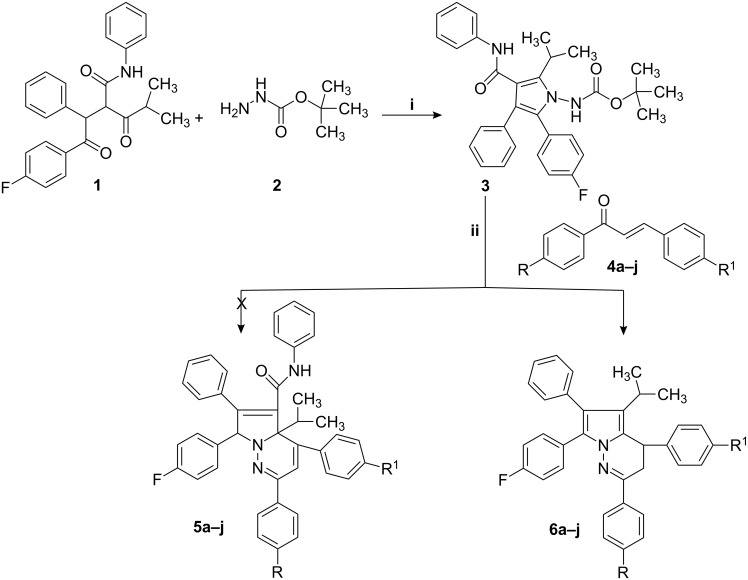
Reagents and conditions: i) *p*-TSA (0.5 equiv), toluene/cyclohexane (4:15), reflux, 15–18 h; ii) *p*-TSA (1.5 equiv), toluene/cyclohexane (30:20), reflux, 15–25 h.

2-[2-(4-Fluorophenyl)-2-oxo-1-phenylethyl]-4-methyl-3-oxo-pentanoic acid phenylamide **1** was reacted with tertiary butyl carbazate **2** in toluene and cyclohexane in the presence of *p*-toluenesulfonic acid (*p*-TSA) at reflux and the resulting [2-(4-fluorophenyl)-5-isopropyl-3-phenyl-4-phenylcarbamoyl-pyrrol-1-yl]-carbamic acid *tert*-butyl ester **3** was further condensed with 3-(4-fluorophenyl)-1-phenyl-propenone **4a** [29] in the presence of *p*-TSA in the same medium. Pyrrolo[1,2-*b*]pyridazine derivatives, i.e. 4,7-bis-(4-fluorophenyl)-4a-isopropyl-2,6-diphenyl-4a,7-dihydropyrrolo[1,2-*b*]pyridazine-5-carboxylic acid phenylamide **5a**, were expected as products in this synthetic sequence (Scheme 1). However, IR, mass, HRMS, and ^1^H, ^13^C, and 2D NMR spectral data of the product confirmed the structure of the product as 4,7-bis-(4-fluoro-phenyl)-5-isopropyl-2,6-diphenyl-3,4-dihydropyrrolo[1,2-*b*]pyridazine **6a** (Table 1).

**Table 1:** 3,4-Dihydropyrrolo[1,2-*b*]pyridazines **6a**–**j**.

**Entry**	**Product**	**R**	**R^1^**	**Yield (%)**	**Time (h)**

1	**6a**	H	F	86	19
2	**6b**	Cl	F	84	18
3	**6c**	Cl	CH_3_	73	21
4	**6d**	Br	CH_3_	70	22
5	**6e**	Cl	Cl	88	15
6	**6f**	Br	F	82	15
7	**6g**	Br	H	68	23
8	**6h**	Cl	H	70	22
9	**6i**	H	CH_3_	65	22
10	**6j**	CH_3_	CH_3_	64	25

**Figure 2:**
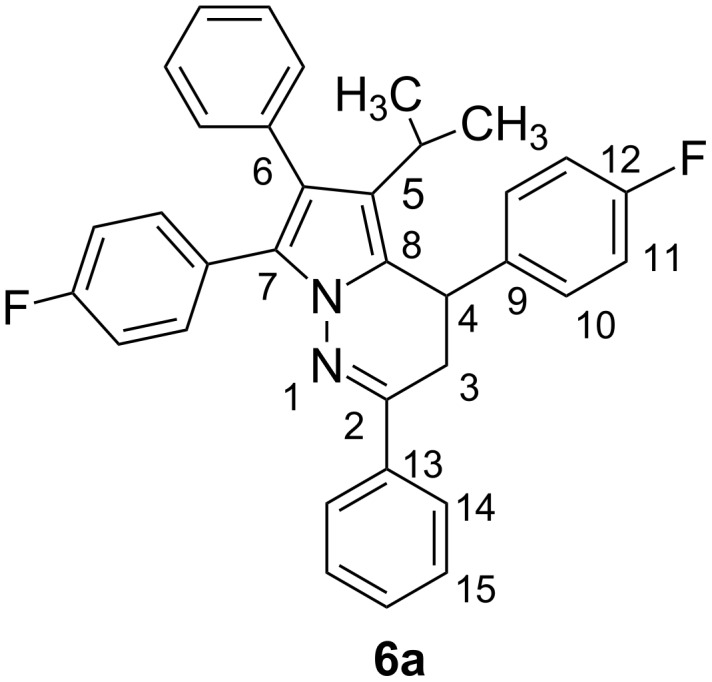
Depiction with proprietary numbering of compound **6a**.

In the mass spectrum of the compound, the molecular ion peak was observed at *m/z* 502 (M^+^), instead of *m/z* 621 (M^+^); HRMS data also confirmed the *m/z* 502 (M^+^) and molecular formula as C_34_H_28_F_2_N_2_, in accord with structure **6a** and not with **5a**. The IR spectrum lacked any –C=O absorption. The ^1^H NMR spectrum of **6a** exhibited signals due to two methyl groups and the –CH of an isopropyl group at δ 0.85 (d, 3H), δ 1.15 (d, 3H), and δ 2.84–2.91 (m, 1H), respectively; –CH_2_ and –CH of pyridazine ring at δ 3.1–3.26 (ddd, 2H) and δ 4.71–4.74 (d, 1H), respectively; and aromatic protons at δ 6.88–7.63 (m, 18H). In the ^13^C NMR, the DEPT spectrum was characterized by the presence of signals due to 2 × CH_3_ and 1 × CH of isopropyl group; CH_2_ and CH of pyridazine ring at δ 22.6, 23.8, and 25.3; 31.5 and 34.7 ppm, respectively. Product **5a** should exhibit a –C=O signal and no CH_2_ peak. In the DQCOSY spectrum, ^1^H–^1^H coupling between 2 × CH_3_ groups and –CH of the isopropyl group and also between the –CH_2_ and –CH groups of pyridazine ring was seen, but no coupling between the isopropyl group and the –CH_2_ and –CH of the pyridazine ring was observed. This indicates that the newly formed –CH_2_ and –CH are connected to each other, which is not possible in **5a**. The HSQC spectrum exhibited ^13^C–^1^H coupling of two methyl groups at δ 23.0, 1.2 (d, 3H); δ 24.0, 0.90 (d, 3H) and –CH of isopropyl at δ 25.5, 2.85 (m, 1H), and also the –CH_2_ and –CH groups of pyridazine ring at δ 31.5, 3.1–3.3 (ddd, 2H); δ 35.0, 4.75 (d, 1H). The newly formed –CH_2_ is linked to a -CH group. It is reported [30] that the 4° carbons of the pyrrole ring resonate at 118 (C-8), 122 (C-6), 124 (C-5), and 132 (C-7). The HMBC spectrum displayed the ^13^C–^1^H correlations of 2 × CH_3_ with only C-5; *^i^*Pr-CH with 2 × CH_3_, C-5, C-6, and C-8; CH of pyridazine ring (C-4) with C-3, C-8, C-5 (less), C-2, C-9, and C-10; CH_2_ (C-3) with C-4, C-2, C-8, C-5 (very small), C-13 (small), and C-9. These data strongly support the linkage of isopropyl group to C-5; C-4 to C-8, C-3; and C-3 to C-2, C-4. All these spectral data are in favor of 4,7-bis-(4-fluoro-phenyl)-5-isopropyl-2,6-diphenyl-3,4-dihydropyrrolo[1,2-*b*]pyridazine structure **6a** (Figure 2), but not of 4,7-bis-(4-fluorophenyl)-4a-isopropyl-2,6-diphenyl-4a,7-dihydropyrrolo[1,2-*b*]pyridazine-5-carboxylic acid phenylamide **5a**.

**Scheme 2:**
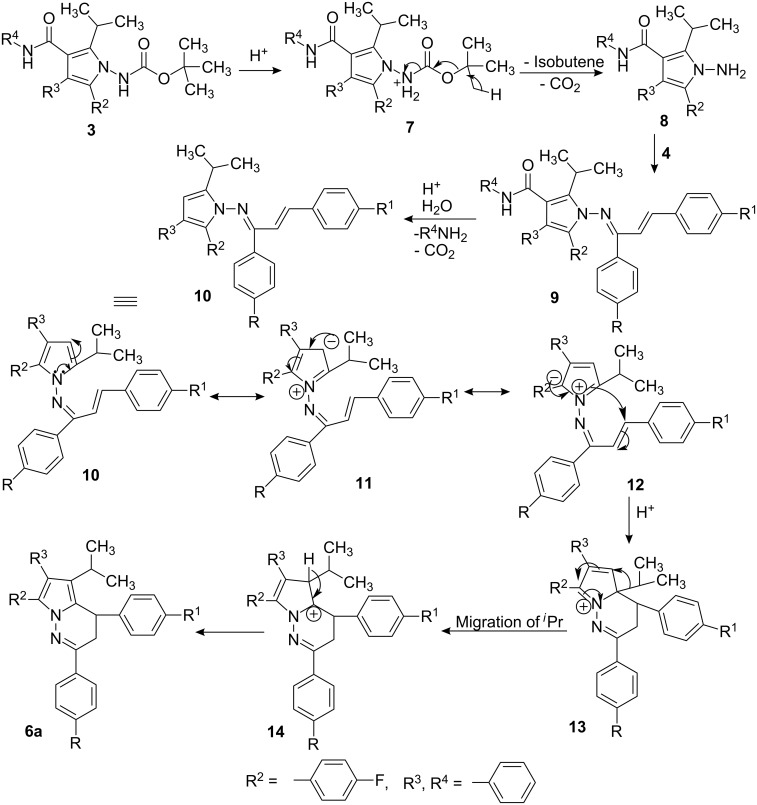
Plausible mechanistic pathway.

A plausible mechanistic pathway for the formation of compounds **6a–j** involves hydrolysis and decarboxylation of carbamate **3**, subsequent condensation with chalcone **4a–j** to provide alkenyl imine **9**, its sequential hydrolysis and decarboxylation, followed by cyclization and migration of the isopropyl group (Scheme 2).

**Scheme 3:**
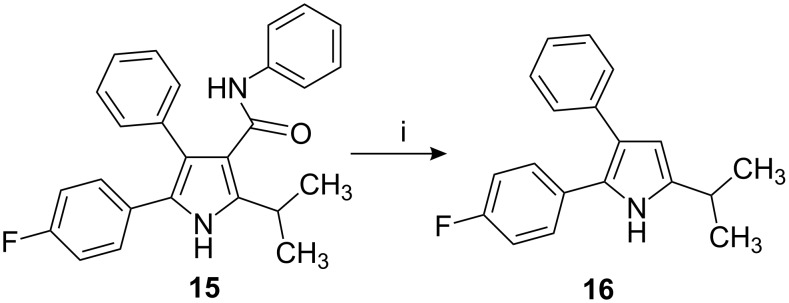
Reagents and conditions: i) *p*-TSA (1.5 equiv), toluene/cyclohexane (1:1), reflux, 10.0 h.

To substantiate the proposed mechanism, the amine **8** was independently prepared from compound **3** by treatment with 33% hydrobromic acid in acetic acid at 30 °C followed by reaction with **4a** in the presence of I_2_ (0.05 equiv) in refluxing ethyl alcohol to provide alkenyl imine **9**, which was characterized on the basis of its mass, ^1^H and ^13^C NMR, DEPT, and IR spectral data. Compound **9** was heated in toluene in the presence of *p*-TSA for 10–12 h and the resulting compound was found to be identical to product **6a**. Hydrolysis of the amide group and subsequent decarboxylation was carried out on pyrrole derivative **15** to afford the 2,3-diaryl pyrrole derivative **16** (Scheme 3).

With a view to extending this protocol to aliphatic systems such as α,β-unsaturated ketones, carbamate **3** was treated with crotonaldehyde under similar conditions. However, the alkenyl imine analogue **9** thus obtained did not undergo further reaction. This may be due to the +I effect of alkyl groups, whereas in the case of aryl groups (−M effect) the olefinic carbon is electron deficient and therefore cyclization is favorable.

**Scheme 4:**
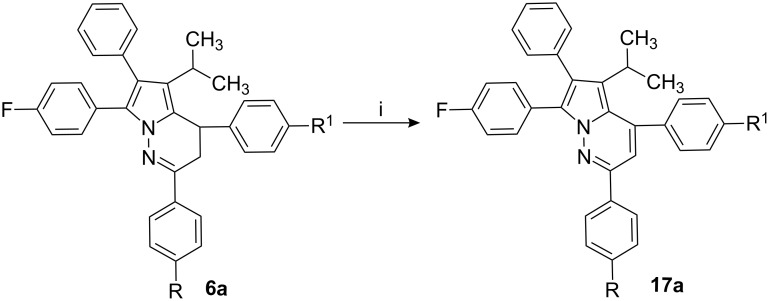
Reagents and conditions: i) *p*-TSA (2.0 equiv), toluene (30.0 volumes).

To aromatize the pyrrolopyridazine ring system, the compound **6a** was heated in the presence of *p*-TSA in toluene at 110 °C for 25.0 h and the resulting compound to yield 4,7-bis-(4-fluorophenyl)-5-isopropyl-2,6-diphenylpyrrolo[1,2-*b*]pyridazine (**17a**, Scheme 4). Other pyrrolopyridazine derivatives **6a–j** were converted into corresponding dehydro derivatives **17a–j** under similar conditions (Table 2).

**Table 2:** Yields and reaction times for compounds **17a–j**.

**Comp No.**	**17a**	**17b**	**17c**	**17d**	**17e**	**17f**	**17g**	**17h**	**17i**	**17j**

R	H	Cl	Cl	Br	Cl	Br	Br	Cl	H	CH_3_
R^1^	F	F	CH_3_	CH_3_	Cl	F	H	H	CH_3_	CH_3_
Yield (%)	75	81	79	76	84	83	63	73	61	59
Time (h)	25	19	23	22	14	14	22	20	28	27

## Conclusion

In conclusion, a facile new approach has been developed for the synthesis of pyrrolo[1,2-*b*]pyridazine derivatives from commercially available and environmentally friendly chemicals. This newly developed method offers quick access to building blocks for various products with pyrrolo[1,2-*b*]pyridazine cores.

## Experimental

The ^1^H, ^13^C NMR spectra were recorded in DMSO-*d*_6_ and CDCl_3_ at 200 or 400 MHz on a Mercury Plus/Varian Gemini 2000 FT NMR spectrometer. Proton chemical shifts (δ) were expressed in ppm with tetramethylsilane (TMS, δ 0.00) as internal standard. Spin multiplicities are given as s (singlet), d (doublet), t (triplet), and m (multiplet). FT-IR spectra were recorded in KBr dispersion with a Perkin-Elmer 1650 FT-IR spectrometer. Mass spectra (70 eV) were recorded on HP-5989 A LC-MS spectrometer. The high resolution mass spectroscopy (HRMS) analysis was performed on the Micromass LCT Premier XE mass spectrometer equipped with an ESI Lack spray source for accurate mass values (Water Corporation, Milford, MA, USA). Melting points were determined by the capillary method with a POLMON (Model MP-96) melting point apparatus. Solvent removal was accomplished by a Buchi rotary evaporator at house vacuum (30–40 Torr). Solvents and reagents were used without further purification. The purity of compounds was checked on silica gel coated aluminium plates (Merck).

**(a) Procedure for compound 3:** A mixture of 2-[2-(4-fluorophenyl)-2-oxo-1-phenylethyl]-4-methyl-3-oxo-pentanoic acid phenylamide (**1**, 5.0 g, 0.012 mol), tertiary butyl carbazate (**2**, 2.06 g, 0.0156 mol), and *p*-TSA (0.006 mol) in toluene (20.0 mL) and cyclohexane (75.0 mL) was maintained at reflux until no more water collected (reaction monitored by TLC). The reaction mixture was cooled to 30 °C, allowed to stand for 3 h, filtered and washed with cyclohexane (10.0 mL). The compound was washed again with cyclohexane (30.0 mL) and dried to give a white solid. mp 191–193 °C; ^1^H NMR (400 MHz, DMSO-*d*_6_, δ ppm): 1.3 (d, 15H, 3CH_3_ of ester and 2CH_3_ of *^i^*Pr), 3.1 (m, 1H, CH), 6.97–7.54 (m, 14H, Ar-H), 9.9 (s, 1H, NH amide), 10.3 (s, 1H, NH ester), both –NH groups were D_2_O exchangeable; IR KBr (cm^−1^): 3422, 3258, 1712, 1671; Anal. Calcd for C_31_H_32_FN_3_O_3_: C, 72.49; H, 6.28; N, 8.18. Found: C, 72.33; H, 6.42; N, 8.35.

**(b) A typical procedure for compound 6a:** A mixture of [2-(4-fluorophenyl)-5-isopropyl-3-phenyl-4-phenylcarbamoyl-pyrrol-1-yl]-carbamic acid *tert*-butyl ester (**3**, 5.0 g, 0.0097 mol), 3-(4-fluorophenyl)-1-phenyl-propenone (**4a**, 2.62 g, 0.0102 mol), and *p*-TSA (2.5 g, 0.0146 mol) in toluene (150.0 mL) and cyclohexane (100.0 mL) was maintained at reflux (azeotropic) for 19.0 h (reaction monitored by TLC). The reaction mixture was cooled to ambient temperature; ethyl acetate (40.0 mL) was added and washed first with water (25.0 mL) and then with 10% sodium bicarbonate solution (25.0 mL). The resulting organic layer was concentrated under vacuum and the crude product recrystallized from ethyl acetate (25.0 mL) to remove unreacted **9** (~5.0%). The resulting filtrate was concentrated under vacuum and further recrystallized from isopropyl alcohol (20.0 mL) to give a cream solid (Table 1). mp 203–205 °C; MS: *m/z* 502 (M^+^); HRMS data: *m/z* 502 (M^+^) and mol. formula: C_34_H_28_F_2_N_2_; ^1^H NMR (400 MHz, CDCl_3_, δ ppm): 0.85 (d, *J* = 7.2 Hz, 3H, CH_3_), 1.15 (d, *J* = 7.2 Hz, 3H, CH_3_), 2.84–2.91 (m, 1H, CH), 3.1–3.26 (ddd, *J* = 6.8, 16.4 Hz, 2H, CH_2_ of ring), 4.71–4.74 (d, *J* = 6.8 Hz, 1H, CH of ring), 6.88–7.63 (m, 18H, Ar-H); ^13^C NMR (100 MHz, CDCl_3_, δ ppm): 22.6, 23.8, 25.3, 31.5, 34.7, 114.0, 114.5, 115.2, 115.7, 118.4, 122.2, 124.0, 126.0, 126.2, 127.0, 127.7, 127.9, 128.3, 128.5, 129.7, 131.2, 132.50, 132.65, 136.1, 136.7, 139.5, 152.3, 159.0, 159.2, 163.8, 164.0; DEPT (200 MHz, CDCl_3_, δ ppm): CH_3_ carbons at δ 22.6, 23.8; aliphatic-CH carbons at δ 25.3, 34.7 and aromatic-CH carbons at δ 114.0–132.6; CH_2_ carbon at δ 31.5 ppm; DQCOSY (400 MHz, CDCl_3_, δ ppm, ^1^H–^1^H coupling): δ 0.8 and 1.2 coupled with δ 2.8 and δ 3.2 coupled with δ 4.8 ppm; HSQC (400 MHz, CDCl_3_, δ ppm, ^13^C–^1^H coupling): δ 23.0, 1.2 (d, 3H, CH_3_ of *^i^*Pr); δ 24.0, 0.90 (d, 3H, CH_3_ of *^i^*Pr); and δ 25.5, 2.85 (m, 1H, CH of *^i^*Pr); δ 31.5, 3.1–3.3 (ddd, 2H, CH_2_ of pyridazine ring); δ 35.0, 4.75 (d, 1H, CH of pyridazine ring); and δ 113–134, 6.9–7.95 (m, 18H, Ar-H); the HMBC spectrum: ^13^C–^1^H correlations of 2 × CH_3_ (124, C-5); *^i^*Pr-CH (2 × CH_3_, C-5, C-6, and C-8); CH of pyridazine ring (CH_2_, C-8, C-5 (less), C-2, C-9, and C-10); CH_2_ (C-4, C-8, C-5 (very less), C-13 (less), C-9, and C-2); IR KBr (cm^−1^): 3064, 1600, 1506, 1155; Anal. Calcd for C_34_H_28_F_2_N_2_: C, 81.25; H, 5.62; N, 5.57. Found: C, 81.41; H, 5.51; N, 5.74.
